# Lessons From Globally Coordinated Cessation of Serotype 2 Oral Poliovirus Vaccine for the Remaining Serotypes

**DOI:** 10.1093/infdis/jix128

**Published:** 2017-07-01

**Authors:** Kimberly M. Thompson, Radboud J. Duintjer Tebbens

**Affiliations:** 1 Kid Risk, Inc, and; 2 University of Central Florida, College of Medicine, Orlando

**Keywords:** polio, eradication, dynamic modeling, disease outbreaks

## Abstract

**Background.:**

Comparing model expectations with the experience of oral poliovirus vaccine (OPV) containing serotype 2 (OPV2) cessation can inform risk management for the expected cessation of OPV containing serotypes 1 and 3 (OPV13).

**Methods.:**

We compare the expected post-OPV2-cessation OPV2-related viruses from models with the evidence available approximately 6 months after OPV2 cessation. We also model the trade-offs of use vs nonuse of monovalent OPV (mOPV) for outbreak response considering all 3 serotypes.

**Results.:**

Although too early to tell definitively, the observed die-out of OPV2-related viruses in populations that attained sufficiently intense trivalent OPV (tOPV) use prior to OPV2 cessation appears consistent with model expectations. As expected, populations that did not intensify tOPV use prior to OPV2 cessation show continued circulation of serotype 2 vaccine-derived polioviruses (VDPVs). Failure to aggressively use mOPV to respond to circulating VDPVs results in a high risk of uncontrolled outbreaks that would require restarting OPV.

**Conclusions.:**

Ensuring a successful endgame requires more aggressive OPV cessation risk management than has occurred to date for OPV2 cessation. This includes maintaining high population immunity to transmission up until OPV13 cessation, meeting all prerequisites for OPV cessation, and ensuring sufficient vaccine supply to prevent and respond to outbreaks.

In 2008, the World Health Assembly resolved to globally coordinate cessation of oral poliovirus vaccine (OPV) following the certification of eradication of wild polioviruses (WPVs) [1]. In 2016 the world started this process with the globally coordinated cessation of serotype 2–containing OPV (OPV2) [2]. Following the 2008 decision, we identified [3] and the World Health Organization (WHO) Strategic Advisory Group of Experts (SAGE) established prerequisites for the Global Polio Eradication Initiative (GPEI) to meet prior to OPV cessation [4]. These prerequisites include interrupting all circulating vaccine-derived polioviruses (cVDPVs) at least 12 months prior to OPV cessation, introducing at least 1 dose of inactivated poliovirus vaccine (IPV) into the routine immunization schedules of all countries, ensuring access to sufficient quantities of licensed bivalent OPV (bOPV) for routine immunization and supplemental immunization activities (SIAs), creating a stockpile of monovalent OPV and a strategy for outbreak response, and developing plans for containment [3, 4]. We also highlighted the need to perform SIAs using trivalent OPV (tOPV) prior to OPV2 cessation to increase population immunity [3].

Heading into the decision by the SAGE to commit to globally coordinated OPV2 cessation during the last 2 weeks of April 2016, the GPEI appeared reasonably on track with respect to most but not all of the prerequisites [5]. The GPEI worked with national immunization programs to ensure IPV introduction and bOPV licensing for routine immunization, which benefited from significant investments in coordination efforts and communication materials. The GPEI developed an outbreak response strategy, commissioned the monovalent OPV (mOPV) stockpile for serotype 2 (mOPV2), and provided an implementation plan and requirements for countries to document their completion of the switch from tOPV to bOPV and removal of any remaining tOPV from their national vaccine infrastructure. The GPEI supported tOPV intensification SIAs in many countries, and thought that it successfully stopped the persistent serotype 2 cVDPVs (cVDPV2s), although 9 confirmed cVDPV2 cases occurred in 3 countries (Nigeria, Guinea, and Myanmar) during the last 12 months prior to the switch [6].

Modeling answered many questions to help the GPEI manage expectations and support decisions and policies related to OPV2 cessation and emphasized the need for aggressive risk management efforts to ensure success. We developed a model of poliovirus transmission and OPV evolution [7, 8] and used it to model the dynamics of OPV cessation without [9] and with [10] IPV. These analyses demonstrated the importance of increasing population immunity to transmission (ie, the ability of all individuals in a population to participate in transmission, taking into account their degree of immunity to symptomatic or asymptomatic infections and how they mix) prior to OPV cessation to ensure die-out of the OPV-related viruses circulating at the time of OPV cessation. The analyses, based on serotype 2 assumptions, further showed the expected die-out of OPV2-related viruses that did not evolve to become cVDPV2s within approximately 6–12 months of OPV2 cessation, the critical role of tOPV SIAs in areas with low population immunity to serotype 2 transmission prior to OPV2 cessation, and the marginal role of IPV in preventing post-OPV2 cessation cVDPV2s. We also characterized the risks for potential poliovirus reintroductions after OPV cessation of all 3 serotypes, including immunodeficiency-associated vaccine-derived polioviruses (iVDPVs) and (un)intentional release risks [8, 11–13]. Modeling of the probability of undetected circulation as a function of time since the last detected case and the type and quality of surveillance demonstrated the importance of high-quality surveillance and intensified tOPV rounds in northwest Nigeria to more rapidly gain confidence in the absence of cVDPV2s [14]. A study of the expected global risks, costs, and benefits of investments in polio risk management for 2013–2052 confirmed the potential benefits of OPV cessation if the GPEI aggressively responds to outbreaks and aggressively manages the risk of low-probability but high-consequence poliovirus reintroduction events that may lead to uncontrollable outbreaks and the need to restart OPV [13]. Modeling studies also explored the potential different timing options for OPV cessation of the 3 serotypes [15], vaccine needs in the run-up to and after OPV2 cessation [16], and the importance of vaccine choices for SIAs prior to OPV cessation [17]. An exploration of different strategies for outbreak response showed that aggressive response with mOPV (or tOPV if any stock remained with the manufacturers shortly after OPV2 cessation) offered the best strategy for controlling and stopping a cVDPV after OPV cessation and avoiding an OPV restart [18]. The same analysis also explored the supply dynamics of the mOPV stockpile and warned of a high risk (ie, 12% for serotype 2) of periods of insufficient mOPV supply to aggressively respond to outbreaks based on a target stockpile size of 100 million filled mOPV doses and a 1-year filling delay [18]. The models showed that using homotypic mOPV (or tOPV) for outbreak response at a level needed to control the initial outbreak did not create a new cVDPV in the outbreak response population, unless used inappropriately long after successfully stopping the outbreak [9], and that using tOPV increased population immunity for serotypes 1 and 3 while also stopping the serotype 2 outbreak [18]. An analysis of the dynamics of the tOPV-bOPV switch showed the risks associated with a nonsynchronous switch and the relative time required for countries to become vulnerable to importations of OPV2, OPV2-related viruses, and cVDPV2s after OPV2 cessation [19]. A separate analysis showed the risks associated with inadvertent tOPV use in routine immunization or SIAs following OPV2 cessation [20].

While many of the model insights extend by analogy from OPV2 cessation to cessation of OPV serotypes 1 and 3 (OPV13), evaluating model insights compared to the actual experience of OPV2 cessation provides an opportunity to better inform risk management for the expected future cessation of OPV13. This study explores the insights and expectations from pre-OPV2 cessation modeling and highlights lessons learned relevant to the planned future cessation of OPV13.

## METHODS

We reviewed the evidence available approximately 6 months after OPV2 cessation and compared the experience thus far with precessation risk model expectations. Specifically, we examined the available data from environmental surveillance and developed a stylized trend representative of the findings available as of November 2016. We simulated the expected die-out of OPV2-related viruses based on the reference case of the global model [13]. The underlying differential equation–based poliovirus transmission and OPV evolution model (ie, the DEB model) characterizes a 20-stage process of evolution from the OPV parent strain (stage 0) to fully reverted vaccine-derived poliovirus (stage 19) with increasingly high basic reproduction numbers (R_0_ values) and paralysis-to-infection ratios [7]. The DEB model assumes the same R_0_ values for fully reverted vaccine-derived poliovirus and homotypic WPVs, with the highest R_0_ values and paralysis-to-infection ratios for serotype 1 followed by serotypes 2 and 3 (Table 1). For WPV and any stage of OPV-related virus, the DEB model simplifies the stochastic and local process of die-out by assuming that no new transmissions can occur once the infectiousness-weighted prevalence drops below a threshold of 5 per million people (ie, the transmission threshold), based on calibration of the model to evidence with WPV die-out and persistence and cVDPV emergence in a diverse set of populations [7]. The global model integrates the DEB model applied to 710 subpopulations of approximately 10 million people with economic inputs, risks of poliovirus reintroductions, and a global mixing structure between the 710 subpopulations, which reflect approximate characteristics related to poliovirus transmissibility, immunization, and surveillance representative of the variability that exists in the world [13]. The global model base case assumed complete and perfect OPV2 cessation by all countries on the same day and that all countries that needed tOPV SIAs prior to OPV2 cessation performed these such that no cVDPV2s would subsequently occur [13]. Our analysis of die-out reports the proportion of all 610 OPV-using subpopulations that still experience OPV2-related virus transmission as a function of time after OPV2 cessation (ie, the proportion in which the prevalence remains above the transmission threshold for at least 1 of the OPV2 reversion stages).

**Table 1.** Serotype-Specific Model Inputs^a^

Model Input	Source(s)	Serotype 1 Estimate	Serotype 2 Estimate	Serotype 3 Estimate
Basic reproduction number (R_0_) for WPV or fully reverted VDPV, relative to serotype 1	[7, 37]	1 (reference)	0.9	0.75
Relative R_0_ of OPV parent strain to WPV or VDPV	[7]	0.37	0.55	0.25
Average time (days) to revert from OPV to fully reverted VDPV	[37]	620.5	408	620.5
Average paralysis-to-infection ratio for fully susceptible individuals	[7]			
WPV		1/200	1/2000	1/1000
OPV		7.4 × 10^–8^	6.2 × 10^–7^	1.3 × 10^–6^
Average per-dose OPV take rate for population modeled in Figure 2	[21]	0.42 (bOPV)	0.60 (tOPV)	0.42 (bOPV)

Abbreviations: bOPV, bivalent oral poliovirus vaccine; OPV, oral poliovirus vaccine; tOPV, trivalent oral poliovirus vaccine; VDPV, vaccine-derived poliovirus; WPV, wild poliovirus.

^a^Excluding small serotype differences in characterization of immunity states [7].

Considering a hypothetical population without seasonality previously used to compare OPV cessation dynamics for all 3 serotypes [21], we explored the maximum time until OPV2-related viruses either die out or cause a cVDPV2 outbreak. We characterize the model results with minimally sufficient population immunity to serotype 2 transmission at the time of OPV2 cessation or minimally insufficient population immunity to serotype 2 transmission at the time of OPV2 cessation, respectively. For both scenarios, we report the average reversion stage of all OPV2-related viruses present in the population to illustrate the expected gradual shift toward more diverged viruses with time after OPV2 cessation.

Recognizing ongoing issues about the perceived risks and benefits of using mOPV for outbreak response, we modeled the implications of avoided mOPV use for outbreak response. The global model base case assumes mOPV outbreak response SIAs (oSIAs) up to 5 years after homotypic OPV cessation on the basis of the assumption that reintroducing large amounts of mOPV viruses longer after OPV cessation comes with unacceptable risks. Specifically, using mOPV for outbreak response at some point after cessation could create new iVDPV excretors or lead to exportation of mOPV-related viruses to areas with insufficient population immunity to prevent their transmission outside of the oSIA target population [13, 18]. Although IPV provides very limited protection from asymptomatic participation in fecal–oral transmission [22, 23], it currently represents the only poliovirus vaccine alternative to OPV. Given the option to use IPV and concerns about mOPV, we further explored the implications of not using mOPV to respond to outbreaks during the first 5 years after homotypic OPV cessation. Specifically, we ran the global model with no outbreak response and with different years after homotypic OPV cessation when oSIAs shift from OPV to IPV (ie, 0, 1, 2, 3, 4, or 5 years of homotypic mOPV use followed by IPV use for outbreak response the remainder of the time horizon). In the model, the decision to use IPV or mOPV for a series of oSIAs occurs based on the time of detection of the outbreak, which means that some oSIAs with mOPV can still occur beyond the cutoff year if detection occurs shortly before the cutoff. For example, for a cutoff to use IPV-only oSIAs at 1 year after homotypic OPV cessation, if an outbreak detection occurs 0.9 years after homotypic OPV cessation, then the oSIAs all still use mOPV, even though the last oSIAs will actually take place >1 year after homotypic OPV cessation. However, in the event that surveillance detects ongoing cases after the last oSIAs in a series, then the next series will use IPV only. The outbreak response strategy during the first 5 years after homotypic OPV cessation for all analyses assumes a 30- or 45-day delay between outbreak detection and initiation of the first oSIA (depending on whether closely connected subpopulations already detected the outbreak), and 4–6 oSIAs per series each reaching 80% of children <5 years of age, with the number and geographical scope depending on the R_0_ in the outbreak population [13, 18]. Other than the vaccine choice, we do not change any of the outbreak response characteristics for the various scenarios we explored, and thus in some cases many repeated large IPV oSIAs occur for which currently insufficient IPV supply exists. Nevertheless, we believe that exploring the implications of not using mOPV provides important context. We focused the analysis on the OPV restart probability, which we estimate based on a weighted sample of global model runs that do (ie, n = 57 runs that represent 5.7% of runs out of a set of 1000 runs) or do not (ie, n = 963 runs that represent 94.3% of runs out of a set of 1000 runs) lead to an OPV restart for the base case [24]. The global model triggers an OPV restart whenever 50000 polio cases accumulate since 2016, which we experimentally determined as an upper bound for the cumulative number of polio cases that typically distinguishes runs with uncontrolled outbreaks from runs in which all outbreaks eventually get controlled [13].

## RESULTS

**Figure 1.**
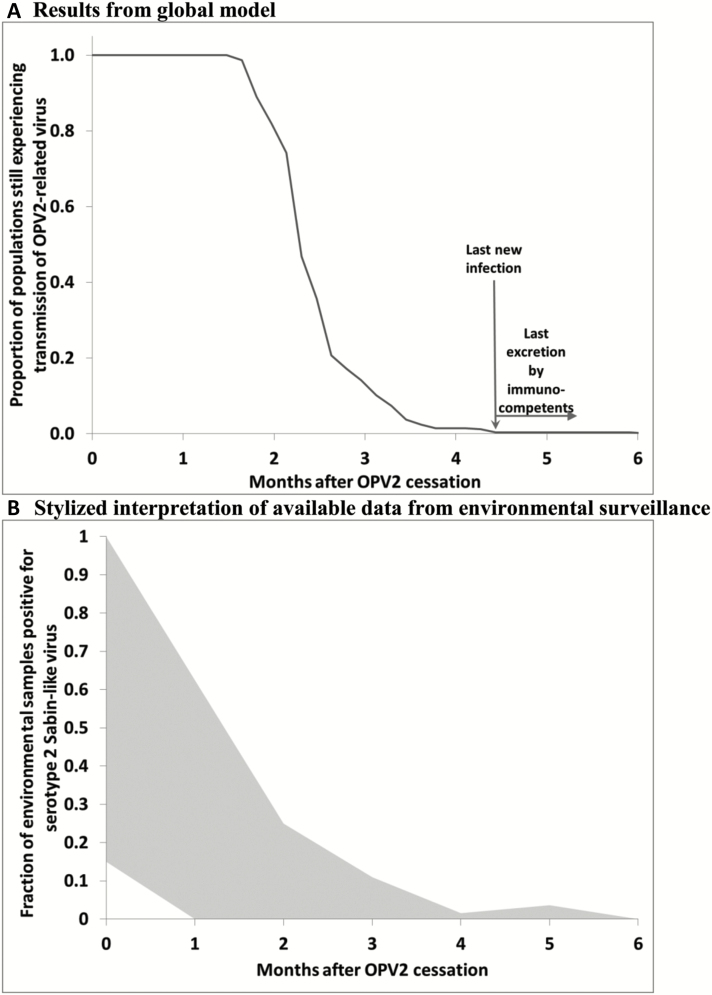
Expectation of oral poliovirus vaccine–related virus die-out based on global model [13] compared to typical pattern observed by environmental surveillance. *A*, Results from global model. *B*, Stylized interpretation of available data from environmental surveillance. Abbreviations: OPV, oral poliovirus vaccine; OPV2, oral poliovirus vaccine containing serotype 2.

Consistent with prior experience of reported observations of ambiguous VDPVs (aVDPVs) [11], the die-out observed in field experiences (eg, Cuba [25–27], New Zealand [28]), and the expectations from prior modeling [29], the results from the global model also suggest relatively rapid die-out of OPV2-related viruses after cessation. Figure 1A shows the expected die-out of OPV2-related viruses after perfectly coordinated OPV2 cessation and sufficient tOPV intensification everywhere [13]. Under those conditions, the global model suggests that transmission of OPV2-related viruses stops a little over 4 months after OPV2 cessation, with excretion by immunocompetent individuals expected to continue for no longer than an additional 2 months (although individuals with primary immunodeficiencies may excrete longer [13, 35]). Observations from environmental surveillance available approximately 6 months after OPV2 cessation suggest die-out within a similar time frame, with most environmental surveillance sites becoming negative for serotype 2 Sabin-like viruses (ie, viruses closely related to OPV2 that did not yet diverge enough to classify as VDPV2s) within 3 months (Figure 1B). Thus, observations to date remain consistent with model expectations. However, we highlight a few notable exceptions. First, Nigeria reported isolation of cVDPV2s from an environmental sample collected in late March 2016, just prior to OPV2 cessation, in the northeastern state of Borno, linked to persistent cVDPV2s from northern Nigeria. The program detected another linked cVDPV2 virus from a healthy contact of a case paralyzed by WPV serotype 1 (WPV1) in Borno in August 2016. Investigations revealed that sustained lack of vaccination and surveillance access in parts of the state not controlled by the central government allowed both the cVDPV2 and WPV1 to continue to circulate without detection for several years. Second, in Balochistan province, environmental surveillance detected multiple VDPV2 viruses. Focusing on bOPV and IPV SIAs to attempt to eradicate WPV1, Pakistan conducted only 1 national tOPV SIA and a few small-scale subnational SIAs in the 12 months leading up to OPV2 cessation, despite a combination of poor SIA quality and low routine immunization coverage in Balochistan province and some other areas that suggested a need to conduct more tOPV SIAs to prevent cVDPV2s after OPV2 cessation [10, 29, 30]. Third, the slight uptick in serotype 2 Sabin-like poliovirus isolation rate at 5 months after OPV2 cessation likely reflects the inadvertent use of tOPV left in the vaccine infrastructure suspected in several high-risk countries. All 3 exceptions represent risks previously highlighted by models (ie, failure to intensify tOPV and inadvertent tOPV use of OPV2 cessation), although neither the GPEI nor models anticipated the extent of the programmatic failure in Borno.

**Figure 2.**
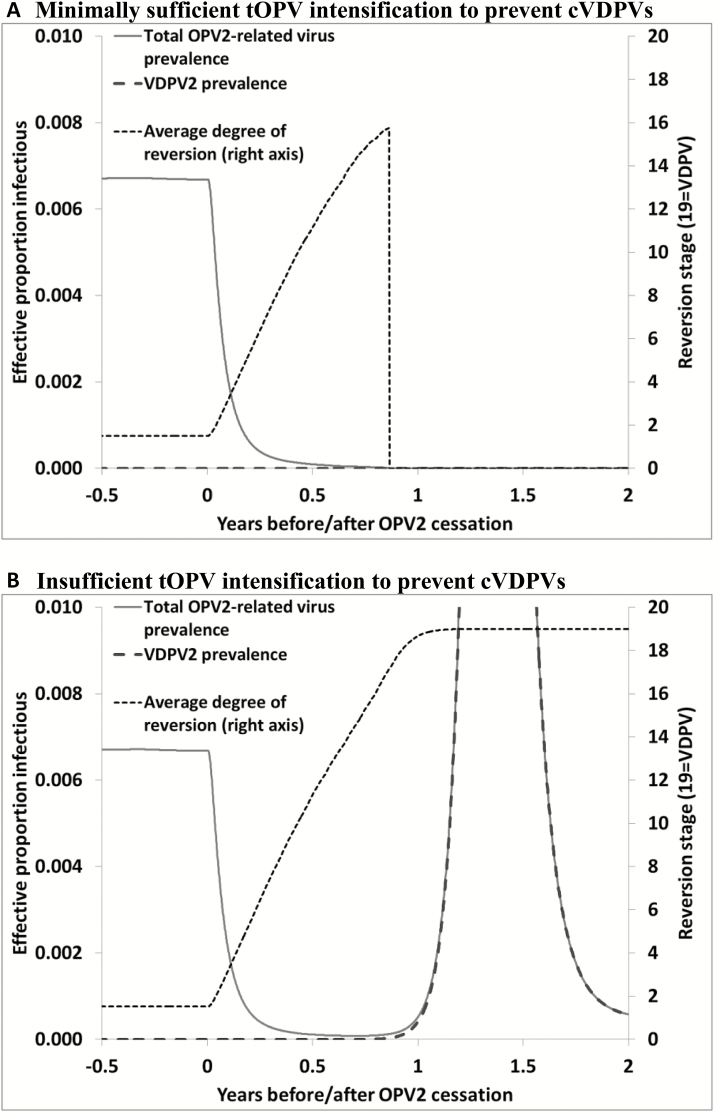
Demonstration of modeled evolution after coordinated oral poliovirus vaccine (OPV) cessation in a hypothetical population without seasonality. *A*, Minimally sufficient trivalent OPV (tOPV) intensification to prevent circulating vaccine-derived polioviruses (cVDPVs). *B*, Insufficient tOPV intensification to prevent cVDPVs. Abbreviations: cVDPV, circulating vaccine-derived poliovirus; OPV2, oral poliovirus vaccine containing serotype 2; tOPV, trivalent oral poliovirus vaccine; VDPV2, serotype 2 vaccine-derived poliovirus.

Figure 2A shows the impacts of minimally sufficient population immunity to serotype 2 transmission at OPV2 cessation as a function of time since OPV2 cessation. The figure shows that OPV2-related viruses may circulate with decreasing prevalence in this hypothetical population without seasonality for almost 1 year (left axis) and evolve to increasing and relatively highly divergent viruses (right axis) after OPV2 cessation even if they eventually die out. These results suggest that detection of isolated partially reverted OPV2-related viruses (ie, aVDPVs) for up to a year after OPV2 cessation may not necessarily indicate a brewing cVDPV2 outbreak. Furthermore, consistent with observations from environmental and acute flaccid paralysis surveillance, we should expect to detect mostly viruses closely related to OPV2 (ie, serotype 2 Sabin-like viruses) during the first months after OPV2 cessation, and expect that any detections further out in time will exhibit more divergence from OPV2 (ie, more nucleotide changes, including VDPV2s). Thus, the longer after OPV2 cessation, the greater the probability that any detected serotype 2 Sabin-like viruses trace to either inadvertent tOPV use or intentional mOPV2 oSIAs. While Figure 2A suggests that OPV2-related viruses that will die out may not die out until almost a year after OPV2 cessation (in the most extreme case), Figure 2B shows that slightly lower population immunity to serotype 2 transmission at OPV2 cessation (ie, slightly less tOPV intensification prior to cessation) may show similar initial behavior shortly after OPV2 cessation, but leads to complete evolution to cVDPV2 and an outbreak instead of dying out. As shown in Figure 2B, in the case of insufficient population immunity to prevent it, transmission and OPV2 evolution continue beyond 1 year after OPV2 cessation due to the availability of more susceptible individuals. Thus, for populations with borderline population immunity to serotype 2 transmission at OPV2 cessation, observations at 6 months cannot definitively establish the ultimate path of either die-out or cVDPV2 emergence.

**Figure 3.**
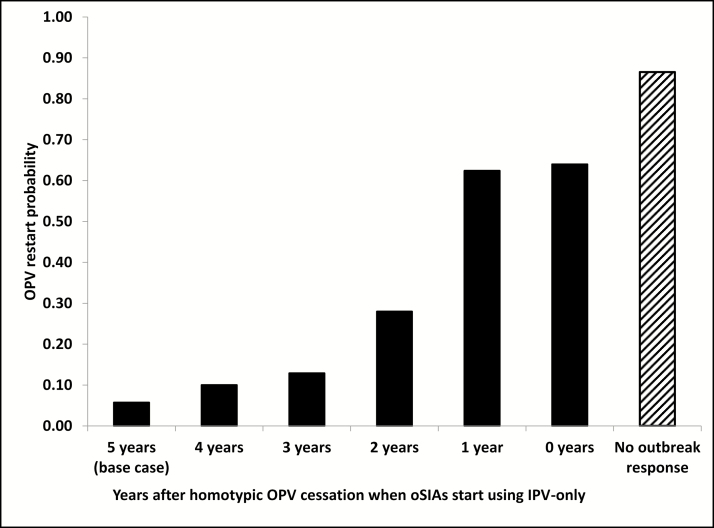
Probability of oral poliovirus vaccine (OPV) restart (ie, uncontrolled outbreaks) as a function of the number of years after homotypic OPV cessation that outbreak response supplemental immunization activities (oSIAs) use monovalent OPV followed by use of inactivated poliovirus vaccine (IPV) and in the absence of any outbreak response.

Figure 3 shows the risks associated with a failure to use mOPV to respond to post-OPV-cessation outbreaks of any serotype. While for the base case that allowed mOPV oSIAs for up to 5 years after homotypic OPV cessation the OPV restart probability remains <6%, this probability increases as we restrict mOPV use for oSIAs. The increase remains relatively minor if we allow mOPV for up to 3 years after homotypic OPV2 cessation, because of the relatively low probability of outbreaks that IPV cannot control occurring >3 years after OPV cessation. However, we find a very significant increase in OPV restart probability (>60%) if we allow mOPV use only for 1 year after homotypic OPV cessation or not at all. Not surprisingly, a failure to respond to outbreaks at all (eg, due to a reluctance to use mOPV or no mOPV available from the stockpile and insufficient IPV supply) further increases the OPV restart probability to >85%. These results suggest that failing to stop an outbreak with a more transmissible and more neurovirulent virus than mOPV represents a much more significant risk than using mOPV to stop the outbreak.

## DISCUSSION

The experience approximately 6 months after globally coordinated OPV2 cessation remains largely consistent with expectations from modeling. Although it remains too early to tell from negative observations whether OPV2 viruses died out, positive signals from Nigeria and Pakistan represent serious risks that the GPEI must aggressively manage. The situation in Nigeria represents a failure to interrupt a persistent cVDPV2 despite the prerequisite to do so before OPV2 cessation [3], and highlights the risk of surprises associated with inaccessible areas in terms of surveillance and vaccination. The situation in Pakistan clearly reflects behavior predicted by models in the event of a failure to intensify tOPV use prior to OPV2 cessation [10, 29, 30]. In both of these situations, WPV1 still circulates and therefore tOPV would represent the optimal vaccine for oSIAs [18]. Unfortunately, the GPEI did not elect to use the sufficient quantities of tOPV still held by the manufacturers and instead destroyed these tOPV stocks in the summer of 2016. The GPEI now finds itself alternating between mOPV2 and bOPV SIAs to respond to both emergencies. Given these concurrent cVDPV2 events and the possibility that further cVDPV2s get detected or iVDPV2 introductions start to cause outbreaks, the supply of filled mOPV2 vaccine from the stockpile may reach critical lows that may limit our ability to respond to events [18]. Although it may seem counterintuitive, using mOPV2 aggressively in response to clear outbreak signals represents the best strategy to avoid further mOPV2 demand and possible stockouts by ensuring that the outbreaks do not establish more widespread transmission. Moreover, serotype 2 Sabin-like virus isolations can provide an indication of inadvertent tOPV use, suggesting the need to ensure complete removal of tOPV from vaccine infrastructures everywhere before these viruses may establish transmission [31]. The GPEI also did not meet the prerequisite of introducing IPV in routine immunization in all countries, in part due to supply issues and in part due to diversion of IPV doses for use in SIAs in endemic countries, although recent modeling suggests this does not represent a cost-effective use of IPV [32, 33].

Prior work suggested that the die-out behavior remains similar for all 3 poliovirus serotypes [21]. However, given different assumptions about the transmissibility and reversion rate of OPV1 and OPV3 compared to OPV2 (Table 1), the population immunity and OPV coverage thresholds needed to prevent cVDPV1 and cVDPV3 outbreaks after OPV13 cessation differ somewhat compared to serotype 2. Specifically, the assumptions used in the model shown in Table 1 imply that safe OPV1 cessation requires lower population immunity to transmission than OPV2 cessation, but higher routine immunization coverage (with bOPV) due to lower take rates and secondary immunity for OPV1 than for OPV2. For OPV3 cessation, both the minimum population immunity level and the minimum routine immunization coverage remain lower than for OPV1 or OPV2 [21].

Comparison of the experience with OPV2 cessation to date and prior expectations from models imply important lessons for ongoing management of the risks associated with OPV2 cessation and future OPV13 cessation risk management. First, maintenance of bOPV SIAs prior to OPV13 cessation represents a critical strategy to minimize cVDPV risks after OPV13 cessation and to support better management of vaccine supply [34]. Second, OPV13 cessation should not occur unless all countries meet the prerequisites, including interruption of all persistent cVDPVs with consideration of the implications of any inaccessible areas on the probability of continued undetected circulation. Further critical prerequisites include IPV introduction everywhere if possible given IPV supply and establishment of large enough mOPV stockpiles, which requires factoring in the long time delays to meet changes in vaccine demand of both OPV and IPV. Meeting the yet unmet prerequisites for OPV2 cessation related to containment and ensuring complete tOPV withdrawal represent critical priorities for the GPEI. Third, mOPV stockpile management must occur in close agreement with outbreak response guidelines. However, the possibility of aVDPVs that will not continue to circulate for up to a year after OPV cessation implies the need to build some flexibility into the outbreak response guidelines while planning for a sufficiently large stockpile to accommodate all possibilities. Fourth, mOPV hesitancy for oSIAs carries serious potential consequences, including a greater risk of OPV restarts, greater risk of creating greater demands for mOPV at a time when its use becomes more risky, and greater risk of future mOPV stockouts. Finally, although it remains too early to observe medium- and long-term risks of outbreaks due to iVDPVs or other potential live poliovirus releases [13, 35], we stress the importance of aggressively managing these risks to avoid OPV restarts. This includes the continued development of polio antiviral drugs, expanded screening for nonparalytic iVDPV excretors [35], efforts to develop new poliovirus vaccines without the risks associated with the current OPV [36], and continued maintenance of poliovirus surveillance activities, which may include strategic expansion of environmental surveillance.

The model results presented in this study carry over the previously discussed limitations of the DEB model and global model [7, 13, 31]. Specifically, we note that the global model may underestimate the risk that mOPV exportation leads to new cVDPV outbreaks elsewhere because it does not explicitly model the behavior at the border of mOPV oSIA target populations and because the kinetics of OPV evolution around the transmission threshold reduce the ability of point introductions with partially reverted viruses to establish transmission. Despite these limitations, we believe that the high probability of OPV restarts associated with a failure to use mOPV for oSIAs provides important context related to implications of not using mOPV.

Ensuring a successful endgame requires more aggressive OPV cessation risk management than occurred to date for OPV2 cessation. This includes maintaining high population immunity to transmission up until OPV13 cessation, meeting all prerequisites for OPV13 cessation, and ensuring sufficient vaccine supply to prevent and respond to outbreaks.

## Notes

***Financial support.*** This work was supported by the Centers for Disease Control and Prevention (cooperative agreement number 1U2RGH001913-01).

***Supplement sponsorship.*** This work is part of a supplement coordinated by the Task Force for Global Health with funding provided by The Bill and Melinda Gates Foundation and the Centers for Disease Control and Prevention.

***Potential conflicts of interest.*** Both authors: No reported conflicts of interest. Both authors have submitted the ICMJE Form for Disclosure of Potential Conflicts of Interest. Conflicts that the editors consider relevant to the content of the manuscript have been disclosed.
